# Scaling up Action Schools! BC: How Does Voltage Drop at Scale Affect Student Level Outcomes? A Cluster Randomized Controlled Trial

**DOI:** 10.3390/ijerph18105182

**Published:** 2021-05-13

**Authors:** Lindsay Nettlefold, Patti-Jean Naylor, Heather M. Macdonald, Heather A. McKay

**Affiliations:** 1 Active Aging Research Team, Centre for Hip Health and Mobility, Vancouver Coastal Health Research Institute, Vancouver, BC V5Z 1M9, Canada; lindsay.nettlefold@ubc.ca (L.N.); heather.macdonald@ubc.ca (H.M.M.); 2 School of Exercise Science, Physical and Health Education, University of Victoria, Victoria, BC V8P 5C2, Canada; pjnaylor@uvic.ca; 3 Department of Family Practice, University of British Columbia, Vancouver, BC V6T 1Z3, Canada; 4 Department of Orthopaedics, University of British Columbia, Vancouver, BC V5Z 1M9, Canada

**Keywords:** scale-up, implementation strategies, effectiveness, children, school, intervention, physical activity, fitness

## Abstract

Action Schools! BC (AS! BC) was scaled-up from an efficacy trial to province-wide delivery across 11 years (2004–2015). In this study we: (1) describe strategies that supported implementation and scale-up; (2) evaluate implementation (teachers’ physical activity (PA) delivery) and student’s PA and cardiorespiratory fitness (CRF) within a cluster randomized controlled trial during years 2 and 3 of scale-up; and (3) assess relationships between teacher-level implementation and student-level outcomes. We classified implementation strategies as process, capacity-building or scale-up strategies. Elementary schools (*n* = 30) were randomized to intervention (INT; 16 schools; 747 students) or usual practice (UP; 14 schools; 782 students). We measured teachers’ PA delivery (*n* = 179) using weekly logs; students’ PA by questionnaire (*n* = 30 schools) and accelerometry (*n* = 9 schools); and students’ CRF by 20-m shuttle run (*n* = 25 schools). INT teachers delivered more PA than UP teachers in year 1 (+33.8 min/week, 95% CI 12.7, 54.9) but not year 2 (+18.8 min/week, 95% CI −0.8, 38.3). Unadjusted change in CRF was 36% and 27% higher in INT girls and boys, respectively, compared with their UP peers (year 1; effect size 0.28–0.48). Total PA delivered was associated with change in children’s self-reported MVPA (year 1; r = 0.17, *p* = 0.02). Despite the ‘voltage drop’, scaling-up school-based PA models is feasible and may enhance children’s health. Stakeholders must conceive of new ways to effectively sustain scaled-up health promoting interventions if we are to improve the health of students at a population level. Clinical Trials registration: NCT01412203.

## 1. Introduction

Physical activity (PA) [1] and cardiorespiratory fitness (CRF) [2] are powerful, independent [3] indicators of child and youth health. Yet, relatively few children and youth engage in recommended amounts of PA [4]; national and international data show secular declines in both PA [5,6] and CRF [6,7,8]. PA and CRF track across childhood [9], into adolescence [10] and early adulthood [11,12]. High CRF in adolescence is associated with reduced risk of cardiovascular events, cancer and premature mortality in adulthood [13,14,15]. Therefore, effective strategies that reach large numbers of young children are urgently needed to promote PA and CRF, and to maintain behaviors across the life course.

Schools are a focal setting for health promotion [16]; ‘whole-of-school’ models are one of eight best investments to enhance PA [17]. Children spend nearly half their waking hours at school, and schools reach diverse populations of children across sociodemographic and socioeconomic backgrounds [18]. However, most interventions do not extend beyond short-term (e.g., one month to one school year in length) well-controlled efficacy studies with relatively small cohorts [19,20,21]. Further, a recent meta-analysis found little evidence that school-based interventions effectively enhanced daily moderate-to-vigorous PA (MVPA) [22]. Although findings could relate to implementation, relatively few studies evaluate this link [20]. Implementation frameworks, designed to guide effective delivery of health promoting interventions, were seldom used [20,21].

“Developing effective interventions is only the first step toward improving the health and well-being of populations” [23]. Effective interventions must be moved out of the research setting, delivered broadly, and sustained within supportive systems to effect change at a population level [19]. Few school-based PA interventions have been scaled-up [19,24]; school-based interventions implemented at scale over longer time periods (e.g., >2 years) are even more scarce [25]. We define scale-up as “the process by which health interventions shown to be efficacious on a small scale and or under controlled conditions are expanded under real world conditions into broader policy or practice” [26].

In 2003, we developed, implemented and evaluated Action Schools! BC (AS! BC) [27] in a randomized controlled efficacy trial involving 10 elementary schools in British Columbia (BC), Canada. We showed that AS! BC was feasible for teachers to deliver, and effectively enhanced delivery of classroom-based PA [28]. AS! BC improved children’s PA [29], CRF [30] and bone health [31]—likely mediated by increased PA delivery by teachers [28]. Importantly, academic performance was not compromised despite children in intervention schools spending more time engaged in PA [32]. More than 90% of teachers were satisfied with AS! BC training workshops, classroom resources and the Support Team. Importantly, 70% of teachers were confident in their ability to deliver AS! BC activities in their classroom following the training workshop [28]. Based on these promising outcomes the BC government supported phased, province-wide scale-up beginning in 2004; AS! BC was sustained—defined here as sustained delivery and stakeholder support—through 2015.

Here we describe AS! BC scale-up across 11 years, and provide results from the two-year randomized controlled effectiveness trial we conducted beginning in the second year of scale-up. We aimed to assess whether the AS! BC model could be delivered at broad scale to schools in a more diverse range of communities and geographies, while maintaining the student-level health benefits we observed in our previous trial [29,30]. While we previously described the ‘comprehensive pathway’ [33] by which we scaled up AS! BC [34], here we heed the call to more clearly describe implementation strategies [35] we adopted to scale-up AS! BC and support implementation at scale for 11 years.

Our objectives are three-fold: (1) to describe strategies that supported implementation of AS! BC across 11 years of scale-up (2004–2015); (2) to evaluate implementation (teachers’ PA delivery) and impact (children’s PA and CRF) within our cluster randomized controlled trial during years two and three of province-wide scale-up; and (3) to assess relationships between teacher-level implementation and student-level outcomes.

## 2. Action Schools! BC

We developed AS! BC in partnership with BC Ministries of Health and Education and describe the model in detail elsewhere [27,34]. Briefly, AS! BC was a flexible, whole of school model that provided elementary schools and generalist teachers with tools and support to create customized action plans. Action plans enabled schools and teachers to provide more opportunities for more children to make healthy choices more often. Overall, AS! BC aimed to increase children’s PA (to 150 min/week) across six Action Zones (School Environment, Scheduled Physical Education (PE), Classroom Action, Family and Community, Extra-curricular, School Spirit). The model was not curriculum based; instead, teachers and schools chose Action Zone(s) they wished to target to enhance PA opportunities for students. ‘Classroom Action’ was the only prescriptive component of AS! BC; teachers were asked to provide an additional 15 min/day of PA during class time (75 min/week) in addition to the weekly schedule of physical education. We describe key components of AS! BC in Table 1.

As per models that describe ‘essential’ elements of implementation and scale-up [36,37], the AS! BC Support Team (JW Sporta; Support Team) was crucial to AS! BC implementation and scale-up success [28]. JW Sporta included experts in healthy living and education who had worked within the BC elementary school system for > 20 years to develop and deliver sport education resources. As the cornerstone of implementation, they established ‘resource teams’ [37] who guided all aspects of implementation and evaluation of AS! BC in schools province-wide. That is, they worked closely with a host of school community partners at regional and provincial levels to develop AS! BC materials, tools and products, and systematically built capacity within schools to deliver the intervention [28]. The Support Team widely promoted AS! BC, worked to build and maintain partnerships with schools and a broad range of local, provincial, national and international stakeholders, and collaborated with the research team to design and conduct the evaluation. During scale-up, the Support Team was comprised annually of 5–8 full and part-time staff and various contractors who worked with ~75 teacher/facilitator trainers annually. Teachers/facilitators who delivered AS! BC workshops had travel and training expenses covered and received either an honorarium or were bought out of teaching time (Teacher on Call) at their own school as needed to deliver training.

**Figure 1:**
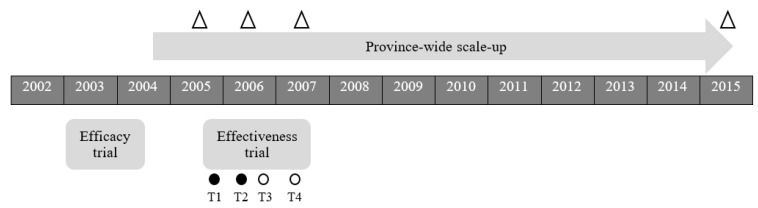
Timeline of Action Schools! BC (AS! BC) scale-up and the randomized controlled effectiveness trial. Open triangles indicate dates where we report implementation strategies and scale-up data (end of first, second, third and final year of scale-up; objective 1). Circles indicate the start of each data collection period within the randomized controlled effectiveness trial (objectives 2 and 3); closed circles represent the beginning (T1) and end (T2) of year 1, open circles represent the beginning (T3) and end (T4) of year 2.

We provide a detailed overview of AS! BC’s ‘comprehensive pathway’ [33] to scale-up elsewhere [34] and provide a timeline in Figure 1. With continued support from BC Ministry of Health, we built on the success of AS! BC, demonstrated in our efficacy study [27,29,30], and initiated provincial scale-up in 2004. AS! BC began as a PA initiative for grade 4–7 students; however, the program was continually enhanced to include Healthy Eating (2006 [38]), PA for kindergarten—grade 3 (2006), PA Student Leadership (2006), and Healthy Eating Student Leadership (2011).

## 3. Materials and Methods

### 3.1. Objective 1: Implementation Strategies across 11 Years of Province-Wide Scale up (2004–2015)

To address our first objective, we retrospectively reviewed the Support Team’s detailed annual reports and monthly statistical summaries. We first generated a list of implementation strategies, defined as “methods or techniques used to enhance adoption, implementation, and sustainability of evidence-based interventions (EBIs)” [39]. We grouped implementation strategies into three broad categories [40]: (a) implementation process; (b) capacity-building and; (c) scale-up. Implementation process strategies are “processes or activities that implementation or quality improvement (resource) teams perform to plan, select, and integrate an EBI into practice” [40]. Capacity-building strategies targeted individuals’ general capacity (motivation, self-efficacy) to execute implementation process strategies (e.g., training and technical assistance) [40]. Scale-up strategies enacted by the support system targeted implementing a specific EBI in multiple settings [40]. We map our strategies to standardized language [39] and follow reporting guidelines [41] to describe each strategy. We extracted data on reach of AS! BC at scale-up (e.g., number of schools registered), and implementation strategies at scale-up (e.g., number of workshops delivered, number of participants attending) from the Support Team’s reports.

### 3.2. Objectives 2 & 3: Randomized Control Effectiveness Trial (2005–2007)

We conducted a cluster randomized controlled trial of AS! BC, that incorporated aspects of a hybrid type I effectiveness-implementation study design [42] in a subset of schools (Clinical Trials Registry NCT01412203) beginning in year 2 of provincial scale-up. A school cluster design was used given the whole-school nature of AS! BC. Evaluation took place over two school years (2005–2007) with measurement scheduled for the beginning and end of each school year (T1–T4; Figure 1).

#### 3.2.1. Study Design and Participants

**Figure 2:**
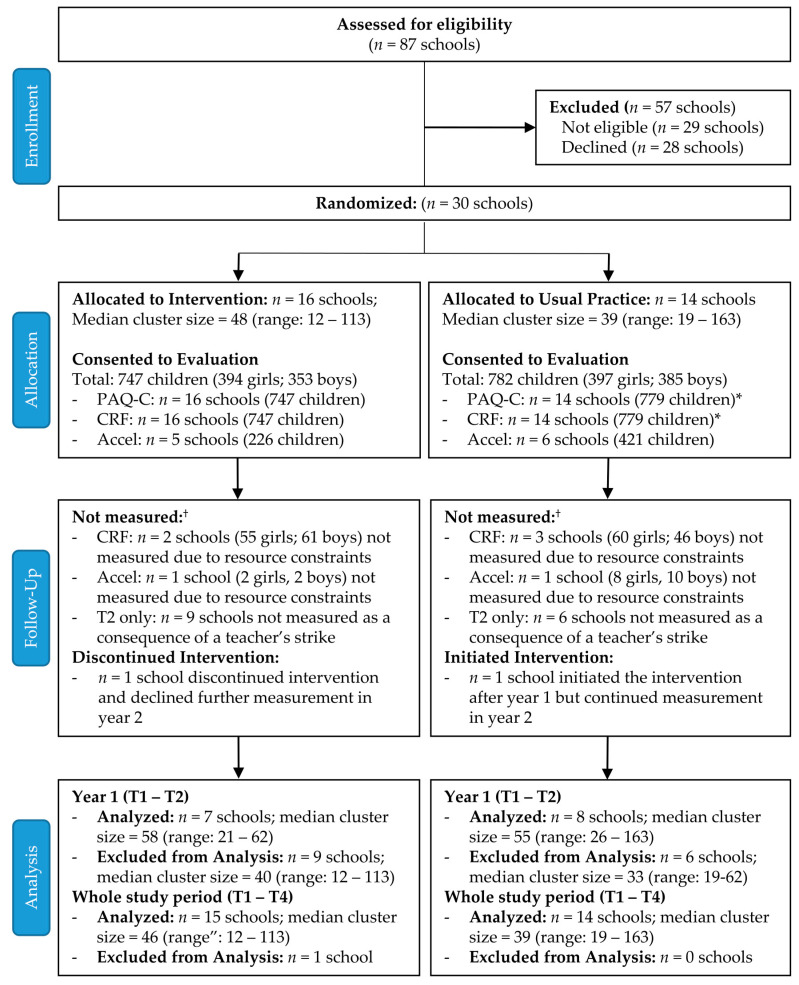
CONSORT diagram illustrating flow of schools through study. * 3 girls at UP schools consented to accelerometry only. † Details on follow-up and analysis at the student-level are described in text and tables for each outcome.

The research team introduced AS! BC and the research study at the annual province-wide principals and vice principal’s meeting in Vancouver, Canada (2005). A formal letter inviting principals to participate was sent to all BC schools. Administrators from eighty-seven schools from across BC expressed interest; we contacted them and 30 schools that met our inclusion criteria (not currently participating in AS! BC or any other PA or healthy eating program beyond physical education (PE)) volunteered to participate (Figure 2). We invited all grade 4 and 5 teachers in volunteer schools to participate in the evaluation. Schools were stratified by size (≥300 or <300 students) and geographic location (5 health regions) and randomly assigned to intervention (INT; *n* = 16 schools) or usual practice (UP; *n* = 14 schools) conditions by an external research group using a computer generated sequence. Schools randomized to the UP group maintained their regular activities.

All grade 4 and 5 students (of participating teachers) who participated in regular PE and provided written informed consent from a parent or guardian, were eligible for the evaluation. Schools incorporated AS! BC into regular programming; all students took part in the intervention, regardless of whether they consented to be evaluated. Research ethics boards at the University of British Columbia (B05-0505) and University of Victoria (07-05-149f) approved this investigation.

#### 3.2.2. Implementation Measures (Physical Activity Delivery by Teachers)

All teachers were asked to complete weekly PA logs to record the PA that their class engaged in; this included PE and additional PA such as Classroom Action (INT schools only) and PA-related field trips. For each teacher, we calculated the percentage of weekly logs returned and average weekly PA delivered (min/week). For teachers at INT schools, we also calculated average weekly Classroom Action PA (min/week) as an indicator of fidelity to the model.

#### 3.2.3. Impact Measures (Student-Level Outcomes)

Trained research staff collected all data; it was not possible to blind research staff to group assignment. Children were excused from their classroom in small groups for measurement (~6/group), which took place in school gymnasia, common spaces, or outdoors

**Self-reported physical activity:** We measured moderate-to-vigorous PA (MVPA) over the previous 7 days in the whole cohort (*n* = 30 schools) using the valid and reliable Physical Activity Questionnaire for Children (PAQ-C) [43], which we modified to include an estimate of time spent in various leisure-time activities in question 1 [44]. Research assistants administered the questionnaire in small groups of 4–6 students. We averaged PAQ-C items to create a general PA score ranging from 1 (low active) to 5 (high active) and obtained an estimate of time (min/day; MVPA_PAQ_) spent in MVPA from question 1. Estimated time in MVPA (min/day) was positively skewed; 42 participants (3% of total observations) self-reported MVPA_PAQ_ ≥ 500 min/day. We limited the maximum possible value to 500 min/day (approximately 3 SD from mean). We included participants who had data for both PA score and MVPA_PAQ_.

**Objectively measured physical activity:** In a subgroup of participants at schools in proximity to our research centre (*n* = 9 schools), we measured PA objectively using ActiGraph GT1M accelerometers (Pensacola, FL, USA) with 15-s epochs. Accelerometers were attached to an elastic belt and positioned at the iliac crest. Children were asked to wear the accelerometer during waking hours for five consecutive days (includes weekdays and weekends), only removing it for water-based activities (e.g., swimming, showering). We excluded data from the day of distribution to eliminate between-school differences in distribution time and any initial reactivity to wearing the monitor.

Prior to processing, each accelerometer file was individually screened for spurious data points and patterns. We used custom software (KineSoft Version 3.3.76, Loughborough, UK) and recommended cutpoints [45,46] to classify activity intensity and included all participants with at least 10 h of data on three or more days (no restrictions on weekday vs. weekend days). We considered periods of continuous zeroes ≥ 60 min biologically implausible and excluded them from our analysis. For analysis we used counts/min as an estimate of total PA, and MVPA (MVPA_Accel_), defined using a cutpoint of ≥2296 counts/min [45,46]. We also dichotomized children into those that achieved/did not achieve an average of ≥60 min/day of MVPA to estimate compliance to recommended PA guidelines [47].

**Cardiovascular fitness:** To assess CRF we used a multistage 20 m shuttle run [48] administered in small groups. We recorded the total number of laps each child completed and calculated age- and sex-specific z-scores [49]—we use both the number of laps completed and the z-scores in the analyses. Five schools (two INT, three UP) in northern BC did not complete CRF measurement due to resource constraints.

**Anthropometry and demographics:** We assessed participants’ height without shoes twice to the nearest millimeter using a portable stadiometer (Seca Model 214, Hanover, MD) and body mass twice to the nearest 0.1 kg using an electronic scale (Seca Model 840, Hanover, MD). If measures of height or weight differed by more than 0.4 cm or 0.2 kg, respectively, we obtained a third measure. We used the mean of the two closest values or the median of three equidistant values in our analyses. Body mass index (BMI) was calculated as weight divided by height squared (kg/m^2^). We estimated maturity offset (years from age at peak height velocity, APHV) using validated equations that include measures of age and height [50]. We determined ethnicity based on parental report of parent and/or grandparents birth place. Most children were white (57%) while remaining children were Asian (25%), North American Aboriginal (10%), and other or mixed ethnicity (9%); this approximates the diversity of the BC population [51].

#### 3.2.4. Sample Size Calculation

AS! BC was powered at the level of teacher implementation (PA delivery) to detect a medium effect size [52] of 0.25 between groups. With a power of 0.80 and α = 0.05 we required a minimum of 64 teachers per condition (128 total; based on a minimum of 3 teachers per school representing 22 schools per condition).

### 3.3. Statistical Analysis

**Objective 1—Implementation strategies:** We describe the number of implementation strategies and where relevant, the number of activities/events within each implementation strategy.

**Objective 2—Implementation measures:** At the teacher level, we assessed differences in PA logs completed by study year (year 1 vs. year 2) and group (INT vs. UP) using Wilcoxon rank-sum tests. We fit linear regression models and adjusted for school cluster (Stata command: vce (cluster)) to compare average PA delivery (min/week) for each year between INT and UP groups. At INT schools, we also compared average Classroom Action PA delivery (min/week) between years 1 and 2.

**Objective 2—Impact measures:** At the student level, we compared baseline characteristics of students attending UP and INT schools (separately for boys and girls) using unpaired *t*-tests, ANOVA or Chi-square as required. A month long, provincial teachers’ strike (with subsequent student dismissal from school) prevented data collection at 15 schools (9 INT, 6 UP) at the end of year 1. We compared baseline descriptive characteristics of students attending schools that were (*n* = 15 schools) and were not (*n* = 15 schools) measured at the end of year 1 using unpaired *t*-tests.

We compared student-level outcomes between INT and UP schools at the end of years 1 (T2) and 2 (T4) of the randomized controlled trial. For all PA measures and CRF (number of laps completed) we fit sex-specific linear regression models, adjusted for baseline (T1) value, age, BMI, ethnicity and school cluster (Stata command: vce (cluster)). We also replaced baseline BMI with maturity offset [50] in all models to account for potential differences in maturity between groups. The model for CRF age and sex specific z-scores included both girls and boys and did not include age as a covariate but was otherwise similar. For MVPA_accel_, we included wear time, but it did not improve model fit and was removed from the final model. We assessed model fit visually using model residuals (normality, linearity and homoscedascity) and identified influential data points using Cook’s D statistics. We report the adjusted mean difference with 95% CI (INT-UP) and effect size of the regression coefficient for the ‘group’ variable (Cohen’s d; stata command: esizereg [53]) as an indicator of the magnitude of the between-group difference [54]. We describe effect sizes of 0.2, 0.5 and 0.8 as small, medium and large, respectively [52]. For descriptive purposes, we also report unadjusted percent change where appropriate. Finally, we examined between-group differences in the percentage of girls and boys achieving PA guidelines at the end of year 1 and 2 using Chi-square tests; we use Cramer’s V as an estimate of the magnitude of the difference and apply the same thresholds as for Cohen’s d above. We analyzed student data as per each school’s initial random assignment (intention to treat; ITT). However, the largest school in the study (UP; *n* = 163 students at baseline) spontaneously adopted the AS! BC intervention at the start of year 2. Thus, for the year 2 (T4) analysis we also conducted a sensitivity analysis excluding this school.

**Objective 3—Link between implementation and outcomes:** We investigated the association between PA delivered (dose) and student outcomes in two ways. First, we used Pearson correlations to describe the association between PA delivered (Total or Classroom Action) and student outcomes at INT schools (no adjustment for school cluster). Second, we dichotomized delivery of Classroom Action PA (the only prescriptive component of AS! BC) into ‘high’ (≥45 min/week; ≥60% of target [23]) and ‘low’ (<45 min/week). We then compared change in student-level outcomes between the two groups using linear regression models, adjusted for school cluster as above. All statistical analyses were performed using Stata version 13 (StataCorp, College Station, TX, USA).

## 4. Results

### 4.1. Objective 1: Implementation Strategies across 11 Years of Province-Wide Scale up (2004–2015)

We used a variety of strategies across three broad categories (implementation process, capacity-building, scale-up [40]) to support implementation and province wide scale-up of AS! BC across 11 years. We specify implementation strategies in Table 2 and provide relevant data below.

#### 4.1.1. Implementation Process Strategies

The Support Team helped schools complete the “4 Steps to Becoming an Action School”: register, take stock (needs assessment), take action (action planning), and report. The Support Team set the following targets for initial provincial scale-up in elementary schools: 160/1568 schools (~10%) registered in AS! BC by the end of year 1 (June 2005), 640 schools (~40%) registered by the end of year 2 (June 2006), and 1120 schools (~70%) registered by the end of year 3 (June 2007). The number of registered schools exceeded targets; by the end of the first year of scale-up (June 2005), 617 schools (39%) across BC were registered (involving 3,236 teachers and administrators, and 85,110 students). By the end of the second year of scale-up (June 2006), 935 schools (60%) were registered (involving 6,693 teachers and administrators, and 170,700 students) with representation across all BC school districts; 75% of school districts had more than 50% of schools registered. By the end of year 3 of scale-up (June 2007), 1,316 schools (84%) across BC were registered (involving 12,971 teachers and administrators, and 331,200 students). By the end of the 11-year implementation (2015), >1400 schools (>90%) across BC were registered (involving >87,500 teachers and administrators and reaching approximately 500,000 students).

#### 4.1.2. Capacity-Building Strategies

Capacity-building activities included workshops delivered by the Support Team to train new teachers and maintain interest and momentum within registered schools (e.g., refresher training or workshops on new topics). During scale-up, the Support Team included 5 full-time staff and 75 volunteer teacher trainers annually. Over 11 years of scale-up, more than 225 AS! BC Trainers were trained.

By the end of the second year of scale-up, the Support Team had delivered 608 workshops with 9593 teachers and administrators participating. By the end of the third year of scale-up, 1212 workshops had been delivered with 18,825 teachers and administrators participating. From 2004–2015 the Support Team delivered 5034 workshops (2591 PA; 1572 Student Leadership; 871 Healthy Eating) to 87,631 teachers and administrators (75,341 for PA workshops) representing 1408 (1395 schools did PA workshops; 89%) or 90% of BC elementary schools.

#### 4.1.3. Scale-up Strategies

Scale-up strategies aimed to increase the number of schools delivering AS! BC. The Support Team used a comprehensive marketing and promotion strategy to achieve full school district representation and strategically inform community stakeholders to increase support and build sustainable, far-reaching networks. The Support Team made presentations to principals and administrators in 40% of school districts during the first year of scale-up; presentations were made to the remaining 60% of districts in the second year. The Support Team also coordinated and/or delivered presentations, displays, and/or promotional materials at events supported by *n* = 51 organizations in year two of scale-up and *n* = 64 organizations in year three. Organizations spanned the following sectors: government (e.g., ministries of health, education), education (e.g., teachers’ associations, parent advisory council, universities), health (e.g., health authorities, regional health units), sport (e.g., sport associations, parks and recreation) and community (e.g., neighbourhood houses, youth organizations).

### 4.2. Randomized Control Effectiveness Trial (2005–2007)

#### 4.2.1. Participants

Over the 2-year study 179 teachers (90 INT, 89 UP) and 1529 children (394 INT girls, 353 INT boys; 397 UP girls, 385 UP boys; 64% of eligible students) from 30 schools consented to participate in the evaluation (Figure 2). Due to teacher and student movement between classes after year 1, 128 teachers (72%) participated during 1 year only (59 in year 1 only, 69 in year 2 only); 51 teachers (28%) participated across both years. Therefore, 110 teachers participated in year 1, and 120 teachers participated in year 2.

We provide baseline student characteristics in Table 3. Age, BMI, maturity offset, PA score and CRF were similar between INT and UP girls at baseline. Girls attending INT schools had higher MVPA (self-reported and accelerometer-measured) and total PA and were more likely to achieve PA guidelines than UP girls. Boys attending INT schools were younger, further from APHV (less mature), and less fit than UP boys. However, MVPA_PAQ_ was lower among UP boys. BMI, PA score, MVPA_Accel_ and the proportion meeting PA guidelines was similar between INT and UP boys. In both girls and boys there was a higher proportion of white vs. Asian students at INT schools compared with UP schools.

Baseline BMI, PA score and CRF were similar between girls who were measured at the end of year 1 and those who were not (due to the provincial teachers’ strike). However, girls not measured at the end of year 1 were younger (−0.1 year, *p* = 0.007) and reported higher MVPA_PAQ_ (+20.8 min/day, *p* = 0.009) at baseline than girls that were measured. Age, BMI, and CRF were similar between boys who were measured at the end of year 1 and those who were not. However, boys not measured at the end of year 1 had higher PA scores (+0.1 units, *p* = 0.03) and MVPA_PAQ_ (+32.2 min/day, *p* = 0.001) at baseline than boys at measured schools. Finally, there were more Asian students, and fewer white students at measured schools compared with non-measured schools (% Asian/white/other: 38/45/17 vs. 7/75/18, *p* < 0.001). This was a function of focusing our limited T2 measurement (due to teachers’ strike) on schools in proximity to our research centre in Metro Vancouver.

#### 4.2.2. Physical Activity Delivery by Teachers

Twenty-three teachers in year 1 and 11 teachers in year 2 did not return any activity logs. A further 20 teachers (1 in year 1 and 19 in year 2) completed their logs retrospectively and 11 teachers (all year 2) completed a mix of on-time and retrospective activity logs that we could not disentangle. On average, teachers with one or more retrospective logs reported delivering more weekly PA than those completing all logs on time (+17.1 min/week; 95% CI 4.2, 30.0). As a result, we include only those with exclusively ‘on time’ logs in these analysis (*n* = 86 in year 1 and *n* = 79 in year 2). These teachers returned a median of 81% activity logs (IQR 57–90%) in year 1 and 77% activity logs (IQR 54–94%) in year 2. Teachers at INT schools returned more logs than did teachers at UP schools (median 86% vs. 70% for INT and UP, respectively).

Table 4 summarizes total PA delivery by year and group assignment for teachers with at least one ‘on time’ PA log (year 1: 78%, year 2: 66%). In year 1, INT teachers delivered more PA as compared with UP schools. In year 2, PA delivery was similar between INT and UP schools. Within the ITT analysis, PA delivery did not differ by year within UP or INT schools. However, when we excluded 5 influential data points (PA delivery well above (178–278 min/week) or below (22.5 min/week) the average (122.5 min/wk)) PA delivery at INT schools decreased from year 1 to year 2 (year 1 vs. year 2: −16.7 min/day; 95% CI −32.0, −1.4). Teachers at intervention schools who participated across both years of the study provided +25.4 min/week more PA during year 2 than teachers who were new in year 2. Overall findings did not change with the sensitivity analysis (data not shown). Within INT schools, teachers delivered a similar amount of Classroom Action PA between years 1 (33.1 min/week; 95% CI 25.3, 41.0) and 2 (25.9 min/week; 95% CI 13.7, 38.1).

#### 4.2.3. Student’s Physical Activity and Fitness

**Self-reported PA (PAQ-C):** PA score was similar (effect sizes <0.1), between INT and UP girls at the end of years 1 and 2 (Table 5). PA score was lower among INT boys as compared with UP boys at the end of year 1, but not year 2 (Table 5); however, effect sizes were small (~0.2). Sensitivity analysis did not change girls’ results; however, boys at INT schools had lower PA score at the end of year 2 than boys at UP schools within the sensitivity analysis (−0.2; 95% CI −0.3, −0.02; (Supplementary Table S1)). For girls and boys, MVPA_PAQ_ did not differ between INT and UP groups at the end of year 1 or 2 within ITT or sensitivity analyses (effect sizes all <0.2). We provide the intraclass correlation coefficient (ICC) for baseline, change from T1–T2, and change from T1–T4 in Supplementary Table S2. Replacing baseline BMI with maturity offset at baseline did not change results.

**Objectively measured PA (accelerometer):** For girls and boys, total PA and MVPA_Accel_ were similar (effect sizes range from −0.16 to 0.05) between INT and UP groups at the end of years 1 and 2 (Table 5). Sensitivity analysis did not change boys’ results; however, girls at INT schools had lower total PA and MVPA_Accel_ at the end of year 2 compared with UP girls (Supplementary Table S1). More INT girls than UP girls met PA guidelines at the end of year 1 (40% vs. 22%, *p* < 0.001, effect size = 0.19); however, achievement of PA guidelines was similar at the end of year 2 (33% vs. 22%, *p* = 0.06, effect size = 0.13). Achievement of PA guidelines did not differ between INT and UP boys at the end of year 1 (48% vs. 55%, *p* = 0.3) or year 2 (48% vs. 51%, *p* = 0.7) (effect sizes < 0.1 for both). We provide the intraclass correlation coefficient (ICC) for baseline, change from T1–T2, and change from T1–T4 in Supplementary Table S2. Replacing baseline BMI with maturity offset at baseline did not change results.

**Cardiorespiratory fitness:** On average, girls at INT schools completed 7.5 more laps (95% CI −0.5, 15.5) than girls at UP schools at the end of year 1 (effect size = 0.48; Table 5). The increase in number of laps corresponded to a 26% increase in girls at UP schools, and a 62% increase in girls at INT schools (unadjusted percent change). On average, boys at INT schools completed 4.7 more laps (95% CI 1.4, 8.0) than boys at UP schools at the end of year 1 (effect size = 0.28; Table 5). The increase in number of laps corresponded to a 23% increase in boys at UP schools, and a 50% increase in boys at INT schools (unadjusted percent change). The between-group difference was <1 lap (effect sizes < 0.1) at the end of year 2 for both girls and boys (Table 5). On average, age- and sex-specific z-scores were 0.33 greater (95% CI 0.2, 0.5) in children at INT schools compared with UP children at the end of year 1 (effect size = 0.36); there was no between-group difference at the end of year 2 (−0.03; 95% CI −0.3, 0.2; effect size < 0.1). Sensitivity analysis did not change the findings (Supplementary Table S1). We provide the intraclass correlation coefficient (ICC) for baseline, change from T1–T2, and change from T1–T4 in Supplementary Table S2. Replacing baseline BMI with maturity offset at baseline did not change results.

#### 4.2.4. Link with Implementation

Dose of Total PA delivered was positively associated with change in MVPA_PAQ_ during year 1 (r = 0.17, *p* = 0.02). Dose (Total or Classroom Action PA) did not correlate with change in any other student-level outcome (Supplementary Table S3). Change in student outcomes did not differ between children in classes that received at least 60% delivery of Classroom Action PA compared with classes exposed to <60% in years 1 or 2 (Supplementary Table S4).

## 5. Discussion

Researchers advocate for whole-of-school [55] and scaled up approaches [19], yet a dearth of effective whole school PA interventions were scaled-up [24]. There are myriad reasons why scale-up does not occur. Not least of which is the considerable support, knowledge and sustained resources needed to do so. Thus, we felt it important to chronicle the phased approach we adopted to implement and scale-up Action Schools! BC across 11 years, with committed support from BC government ministries and a host of school community partners (Figure 1). We also describe implementation and scale-up strategies that facilitated implementation and sustained AS! BC delivery, as this may aid other teams working in this important field of study.

Schools are complex and dynamic systems [56], and present many challenges to scaling-up effective interventions. The rapid uptake of AS! BC in the first three years of scale-up illustrated the need and appetite for the intervention, a window of opportunity [57] afforded by the political climate and the success of our scale-up and implementation strategies. It was not possible to formally evaluate the influence of individual strategies. However, we note that our implementation and scale-up approach incorporated many ‘essential elements’ of comprehensive school health implementation models [58]. Our approach also aligned with implementation and scale-up frameworks [23,36,37], and what policy-makers deemed the drivers of successful scale-up and sustainability [57]. Governance, leadership, resources, outsourcing delivery, accountability structures and committed stakeholder engagement all played a key role to support scale-up and sustainability of AS! BC.

Much has been written about the key role of central support teams to build capacity and provide ongoing support to increase deliverers awareness, knowledge, skills, self-efficacy, and motivation to adopt and implement effective interventions [36,59]. Capacity-building studies in public health and community-based practice identified technical assistance, training and tools as central strategies to support effective implementation [23,36,60]. These were all focal points of AS! BC, and received sustained investment from BC government ministries. For example, grants to support scale-up funded the Support Team (5–8 full and part-time staff and various contractors; their office space; travel; communications, etc.), covered costs for teachers and facilitators who delivered AS! BC workshops (travel, training, release time or honoraria as needed), AS! BC resources such as equipment (Classroom Action) bins, resource manuals and other printed materials required for day-to-day delivery of the model, and release time for classroom teachers to attend training workshops.

Unique features of AS! BC also likely played a role in successful scale-up. The multi-component model provided schools flexibility to create their own Action Plans, based on teachers’ and schools’ available resources and self-identified needs. This provided schools autonomy to adapt the model to context—a core condition for successful implementation of a comprehensive school health approach [58]. New implementation strategies and program materials were added over time to address the changing needs of schools, and sustain interest. For example, after the effectiveness trial, the Support Team added new workshops on healthy eating and PA across all grades, arranged teacher mentorship, provided refresher and student leadership training and offered additional resources such as e-newsletters, posters and instructional manuals. The Support Team tracked delivery of each of these; we did not assess outcomes related to these additional activities.

Below, we delve further into the unique aspects of AS! BC scale-up, and present key strengths, limitations and implications of our findings.

Findings from the subset of schools that participated in the two-year randomized, controlled effectiveness trial showed that teachers at intervention schools delivered more PA during year 1 of the study as compared with teachers at usual practice schools. However, teacher compliance with activity logs was a challenge. Others noted this previously and attributed poor compliance to the substantial demands on teachers across a broad range of administrative and instructional tasks [61]. Despite greater PA delivery by teachers in year 1, students’ PA did not increase significantly. This is consistent with some [22,62], but not all [63,64], systematic reviews published in the last 10 years. Even when improvements in students’ PA were observed in other trials, the effect was modest. For example, across 20 controlled trials students’ PA (moderate and vigorous) increased by approximately one min/day [63].

Nevertheless, in the current study, for intervention schools we observed a 36% and 27% (unadjusted percent change) greater increase in girls’ and boys’ fitness, respectively, compared with peers attending usual practice schools during year one. While this was not statistically significant after controlling for the relatively small number of variably sized clusters, we recognize the limitations of relying solely on p values [54]. This between-group difference corresponds to approximately 30–60 s additional running or a difference in peak oxygen consumption of approximately 1.0 mL/kg/min for boys and 1.9 mL/kg/min for girls at the end of year one–two to four times greater than that observed in a recent pooled analysis of 20 controlled trials [63]. We consider the magnitude of change indicated by the meaningful effect sizes, particularly in girls, an important finding. On average, girls are less fit compared with boys—a difference which increases with age and maturation [65]—and, tend to benefit less from interventions than boys [63]. We also consider this result promising in light of the suggested secular decline in children’s fitness of 7.3% between 1981 and 2014 [7]. While long-term implications and clinical relevance of small improvements in children’s CRF are difficult to discern, evidence in adults suggests that even small improvements in CRF yield clinically important outcomes [66]. For example, in adult men, a 1-minute increase in treadmill test duration over approximately 5 years was associated with a 7.9% reduction in all-cause mortality and an 8.6% reduction in cardiovascular disease mortality [66]. As CRF tracks through childhood [10] and adolescence [11] there is a potential for these small changes to influence adult health.

Our previous efficacy trial improved PA [29] and CRF [30] among students who attended AS! BC schools. This pattern, wherein benefits decrease with the move from efficacy to real world trials, is not unique to our work [67]. Scale-up requires adaptation to fit new contexts and delivery systems, and to accommodate resource constraints. This may lead to an attenuation of the intervention effect—known as the “scale-up penalty” [68,69] or an intervention “voltage drop” [70] of 25–50% [67]. In the effectiveness trial, intervention teachers delivered ~14–33 min/week more PA than teachers at usual practice schools; the difference in PA dose between teachers at intervention compared with usual practice schools was higher (45–55 min/week) in our efficacy trial [34]. In the Classroom Action zone, teachers in the effectiveness trial delivered ~47% of the target PA in Year 1, and ~35% of the target in Year 2. An estimated 60% of program delivery is required to elicit beneficial change [23]. Therefore, at AS! BC scale-up, dose of PA delivered may have been insufficient to elicit similar improvements in children’s PA and fitness, as compared with the efficacy trial. Our findings highlight the challenge of implementing an ‘effective’ intervention to accommodate scale-up. This ‘tug-of war’ [71] to retain implementation fidelity while adapting school-based interventions to achieve ‘best fit’ for diverse geographic regions and school populations, is a topic that deserves further attention.

A number of factors may have contributed to voltage drop at scale-up in our study. First, due to a provincial teacher strike, we were unable to collect data in some schools at the end of year 1. This reduced our sample size for key outcomes and impacted our analyses. Second, we decreased the amount of support provided to teachers during the effectiveness trial (as compared with the efficacy trial), as the Support Team sought to reduce intervention delivery costs during scale-up. Third, intervention teachers delivered less PA in year 2 compared with year 1. This might reflect that training sessions in year 2 were ‘refresher’ workshops (~1 h duration) compared with full workshops in year 1 (~3 h duration). Thus, new teachers in year 2 may not have been as well-trained or committed to deliver the AS! BC intervention as teachers in year 1. Teachers at intervention schools who participated across both years of the study provided +25.4 min/week more PA during year 2 than teachers who were new in year 2. We [20] and others [23] have shown a positive relationship between dose and at least one health outcome. We noted a positive association between total PA delivered by teachers and change in students’ PA score over the first year of the study. This highlights that engaged, motivated, and trained teachers are key to successful outcomes at the student level. Thus, as a means to continually build capacity, teacher training through ongoing (new and refresher) courses each year is a worthwhile investment. Fourth, unlike the efficacy trial, there were no self-identified champions at schools during scale-up. Scale-up models [72] identify the key role that local champions play in scale-up success.

### Strengths and Limitations

We highlight several strengths of our study that address gaps in the current literature. Specifically, (i) we describe the chronology of AS! BC scale-up, and implementation strategies that supported its translation into a “real-world” setting. Across school-based health promoting interventions more broadly, only 5 examined sustainability more than 5 years later [25]. To our knowledge, AS! BC is one of only two other whole-school PA models (CATCH [73] and Take10! [74]) to be implemented at scale with continuous stakeholder support over more than a decade; (ii) we evaluated the impact of AS! BC across two years of scale-up in a large and diverse cohort of children (~1500 children across the province); and (iii) we investigated the link between program implementation and student-level health outcomes.

There are many challenges associated with conducting pragmatic, real-world trials in schools. Therefore, we acknowledge several limitations of our study. First, due to variability in the number of children per school (cluster), and a teacher strike that rendered us unable to measure 50% of participating schools at the end of year 1, we could not apply multilevel modeling techniques to account for our clustered study design [75]. However, we adjusted the standard errors to account for the non-independence of individuals from the same school cluster [76]. This approach may have limited our ability to detect statistically significant intervention effects, despite large changes in CRF (Type 2 error). Second, due to poor compliance with activity logs we could not determine the extent to which teachers participated in all aspects of intervention delivery (i.e., across all Action Zones). Third, without direct observation of classroom teachers, and subsequent student participation (not feasible in scale-up studies), we do not know the intensity of PA delivered (dose delivered), or the degree to which students responded to, and were engaged in, the activities (dose received). We recognize the distinction between ‘dose delivered’ by teachers and ‘dose received’ by students and aspects of implementation that influence these implementation indicators (e.g., fidelity, quality and participant responsiveness) [23], that we did not measure.

## 6. Conclusions

It is possible to scale-up and sustain whole of school PA interventions over the longer term (>10 years), with the ongoing support of government and school-community stakeholders. Despite many challenges to doing so, there is a need for implementation resource teams to conceive of new ways to sustain benefits of scaled-up school-based health promoting interventions to improve students’ health at a population level. Support units with established connections to schools and comprised of researchers, government stakeholders and school community-based practitioners are a critical element of scale-up success. Greater PA delivery by trained teachers and the magnitude of change in fitness we observed in AS! BC intervention schools, suggests that scaled-up school-based PA models may enhance children’s health. Notably, implementation and capacity-building through ongoing teacher training, support and compliance are key factors to realize and sustain benefits. Future school-based interventions guided by rigorous implementation process models that support program delivery, and evaluation frameworks that assess key implementation, scale-up and sustainability indicators would be a welcome addition to the current literature.

## Figures and Tables

**Table 1:** Key components of the Action Schools! BC (AS! BC) model. Reprinted from Journal of Science and Medicine in Sport 9(5), Naylor et al [28], Lessons learned from Action Schools! BC—An ‘active school’ model to promote physical activity in elementary schools, p413-423 (2006), with permission from Elsevier.

Component	Description
Action Zones	Six areas in which opportunities for physical activity could be provided to students. The six Zones were: (1) School Environment, (2) School Spirit, (3) Physical Education, (4) Extra-curricular, (5) Family and Community and (6) Classroom Action.
AS! BC Support Team	A central technical support unit that developed and provided AS! BC resources (training workshops, written materials, Classroom Action Bins, school newsletter inserts for families) and ongoing consultation (on-site and telephone) to administrators, teachers and the School Action Team.
AS! BC School Facilitators	Two elementary school teachers seconded by the AS! BC Support Team to provide training, support and advice to the schools and liaise between the Support Team and the School Action Team.
School Action Team	A committee of school stakeholders (e.g., interested intermediate grade teachers, administrators, parents, health, sport/recreation practitioners) that created and supported implementation of the Action Plan.
Planning Guide for Schools and Teachers	A set of inventories and worksheets that guided teachers and the School Action Team to identify school priorities and create their Action Plan.
Action Pages!	A resource directory using curriculum organizers to link teachers, coaches or community instructors with recommended and available resources.
Classroom Action Bin	A storage bin for the classroom filled with playground balls, videos, skipping ropes, exercise bands, strength grippers and teaching resources that supported the Action Plan.

**Table 2:** Scale-up and implementation strategies fell into three categories: implementation process, capacity-building, and scale-up [40].

Strategy	Description	Specification	Alignment with Taxonomy [39]
**Implementation Process Strategies:** The Support Team worked with schools to support implementation of AS! BC			
4 Steps to Becoming an Action School	The Support Team worked with schools on the “4 Steps to Becoming an Action School”: (1) registration (2) taking stock (needs assessment covering 5 areas—equipment, human resources, community resources, teaching resources and school health) (3) taking action (action planning; schools create an Action Team, develop an Action Plan and schedule a workshop) (4) reporting (schools reflect on their Action Plan and progress over the school year)	**Actor:** AS! BC Support Team **Action:** Once a school indicated interest the Support Team sent materials (detailed below) to assist the school. Each school completed a needs assessment (see ‘Taking Stock’, in adjacent column) and created an Action Plan that supported the school’s goals and aligned with available resources. The flexibility of the AS! BC model made it adaptable to schools needs and available resources. Schools completed year-end reporting related to progress on the Action Plan. Action plans and annual reports were received and reviewed by the Support Team. **Target:** Participating schools **Temporality:** Needs assessment and action planning were done after registration. Reporting occurred annually at the end of the school year.	Provide ongoing consultation Provide local technical assistance Conduct local needs assessment Assess for readiness and identify barriers and facilitators Promote adaptability
Teacher on Call Provincial release time	The BC Government provided funding for a Teacher On Call to allow at least one teacher per intervention school to attend an AS! BC workshop, or participate in another activity to support implementation of AS! BC at their school.	**Actor:** AS! BC Support Team **Action:** Release time **Target:** Participating schools/teachers **Temporality:** Once after registration and again as new training opportunities were offered (e.g., as AS! BC expanded to include Healthy Eating, release time was available to schools again)	Access funding
AS! BC Classroom Action Bin	Participating schools received a Classroom Action Bin (1/grade). Bins included equipment and best-practice resources teachers could use to support their Action Plan (e.g., playground balls, DVDs/videos, skipping ropes, posters promoting movement).	**Actor:** AS! BC Support Team **Action:** The Support Team provided Classroom Action Bins to support schools implementing AS! BC. **Target:** Classroom teachers **Temporality:** Resources were available on the website. The Support Team regularly updated materials based on feedback from participating schools, administrators and teachers. Action bins were provided (1/grade) once schools created an action plan.	Provide equipment
AS! BC support and communication materials	The Support Team developed materials to help schools promote healthy living (including PA and healthy eating) within the school community. Key resources included: AS! BC planning guide (‘how to’ manual that guided schools and teachers through the steps to become an ‘Action School’); Classroom Action resource (support delivery of Classroom Action—additional 15 min PA/day); Introduction to Classroom Action Zone DVD (1/school); Action Pages! (Nationwide inventory of PA, PE and healthy eating resources); AS! BC playground circuits (customized for schools and made available on the website); Weekly activity logs to track PA delivered to students; E-news delivered to administrators and teachers; Success stories shared on website and through outreach channels.	**Actor:** AS! BC Support Team **Action:** The Support Team developed, updated (e.g., revisions, new materials) and distributed support and communication materials to support schools implementing AS! BC. **Target:** Schools and teachers **Temporality:** Resources were available on the website. The Support Team regularly updated materials based on feedback from participating schools, administrators and teachers. E-news was delivered monthly.	Provide equipment Develop and distribute educational materials Obtain and use feedback Capture and share local knowledge
Branded incentives	Additional resources and equipment to support PA and healthy eating in the school.	**Actor:** AS! BC Support Team **Action:** The Support Team offered incentives to schools to encourage them to complete annual reports. **Target:** Participating schools **Temporality:** Annually to support reporting and also periodically throughout the year	Provide incentives
Advisory committees	The provincial advisory committee had community, school and government representatives. The school advisory committee had teacher and principal representatives	**Actor:** Principal investigators and AS! BC Support Team **Action:** After receipt of funding, the committees were formed to provide input on design and implementation of AS! BC **Target:** Key stakeholders **Temporality:** Advisory groups met regularly throughout delivery	Use advisory boards and workgroups Create an implementation blueprint
Teacher Mentorship	Additional support for schools and teachers	**Actor:** AS! BC Support Team **Action:** Trainers were available to provide additional support including classroom visits, action planning, planning for healthy eating, organizing equipment and resources, etc. **Target:** Participating schools and teachers **Temporality:** As requested throughout delivery	Provide ongoing consultation
**Capacity-building Strategies:** involve the management and implementation of a process to deliver AS! BC workshops, build capacity across the province, and maintain interest and momentum within registered schools.			
AS! BC workshops and ongoing support	Training workshops were coordinated by the Support Team and delivered by the Support Team, Master or Regional Trainers. Ongoing support (via email, phone) was available as needed.	**Actor:** AS! BC Support Team **Action:** The Support Team worked with schools to secure a date for workshops. Classroom Action Training workshops were 3 h long. Refresher training workshops were 1 h long. Training was delivered face-to-face to groups of teachers in schools (gymnasia, multi-purpose rooms, empty classrooms) and followed principles of experiential learning and self-efficacy theory. Training workshops included positive modelling, verbal persuasion and opportunities for teachers to be successful and recognized. The Support Team and Trainers provided ongoing support during the school year via email and telephone, and over time teachers/schools could register for a selection of additional in-person workshops throughout the year (e.g., student playground leadership). Workshop participants completed evaluation forms, which were returned to the Support Team and used to improve future workshops. **Target:** Participating teachers and schools **Temporality:** Ongoing throughout the year	Conduct ongoing training Make training dynamic Obtain and use feedback
AS! BC Master and Regional Trainers	The Support Team used a network of Master Trainers and Regional Trainers to deliver workshops across the province. Regional trainers self-identified as interested and submitted a resume. If accepted, they participated in training during a Summer Institute, offered annually.	**Actor:** AS! BC Support Team **Action:** The network of Master and Regional Trainers supported the delivery of workshops province-wide. **Target:** Participating teachers and schools **Temporality:** Ongoing throughout the year	Identify and prepare champions Train the trainer
Summer Institute	The Summer Institute served as a training opportunity for Master and Regional trainers.	**Actor:** AS! BC Support Team **Action:** Individuals selected as Master or Regional Trainers attended the Summer Institute led by the Support Team. The Summer Institute used a train the trainer approach to build training capacity across the province. **Target:** Future Master and Regional trainers **Temporality:** The Summer Institute was 2 days in duration held in August prior to the start of the school academic year.	Identify and prepare champions Make training dynamic Train the trainer
Regional Trainer Support Materials	A number of resources supported the Master and Regional trainers. Key resources included: Regional Trainer Guides (workshop templates, presentation tips, checklists, evaluation forms and handouts for schools); Regional Trainer bags (teaching resources and equipment for leading workshops); AS! BC branded materials (e.g., clothing, clipboard, water bottle, pens); E-news	**Actor:** AS! BC Support Team **Action:** The Support Team provided Master and Regional Trainers with materials to support workshop delivery around the province **Target:** AS! BC Master and Regional Trainers **Temporality:** Master and Regional Trainers received support materials prior to delivering workshops. E-news was delivered monthly.	Provide equipment
AS! BC website	The AS! BC website was updated regularly with registration numbers and contained resources for teachers and schools including: playground circuits, and the latest versions of all support materials and teaching resources.	**Actor:** AS! BC Support Team **Action:** The Support Team kept the website up to date with the latest registration data (by school district and school) and resources. **Target:** Teachers, schools, communities **Temporality:** Updates and additions to website were ongoing	Develop and distribute educational materials
**Scale-up Strategies:** The AS! BC Support Team used a comprehensive marketing and promotion strategy to achieve full school district representation and strategically inform community stakeholders to increase support and build sustainable, far-reaching networks for the initiative.			
Build partnerships	To build partnerships with schools and stakeholders the Support Team participated in a number of events. This was an opportunity to promote AS! BC, network with relevant stakeholders. Stakeholders spanned the following sectors: government (e.g., ministries of health, education), education (e.g., teachers’ associations, parent advisory council, universities), health (e.g., health authorities, regional health units), sport (e.g., sport associations, parks and recreation) and community (e.g., neighbourhood houses, youth organizations).	**Actor:** AS! BC Support Team **Action:** Coordinated and/or delivered presentations, displays or promotional materials at events to support networking, relationship building, and ongoing collaboration with key stakeholders **Target:** Community and provincial organizations within BC, across Canada, and internationally **Temporality:** Ongoing during scale-up	Promote network weaving Work with educational institutions
Recruitment strategies	A range of marketing and promotion strategies were used to recruit teachers, school administrators and other members of the school community to be involved with AS! BC.	**Actor:** AS! BC Support Team **Action:** Prepared and shared one-pagers, rack cards, brochures, promotional posters, AS! BC displays, media profiles, mailouts to schools and managed the AS! BC website. The Support Team responded to requests for information (from website) and used targeted approaches (e.g., contacting schools directly) to engage schools. **Target:** Teachers, school administrators and other members of the school community **Temporality:** Mail-outs occurred twice a year (Sept, Jan). Other activities as needed and ongoing during scale-up.	Develop and distribute educational materials Increase demand
Promotional Strategies	The Support Team used a variety of promotional strategies to inform schools and community stakeholders about AS! BC.	**Actor:** AS! BC Support Team **Action:** The Support team made presentations to principals and administrators at school district meetings, and to teachers at school staff meetings. At these meetings, the Support Team provided an overview of AS! BC including requirements and benefits of enrolling. Other promotional strategies included maintaining an AS! BC website and semi-annual mailouts to all BC elementary and middle schools. **Target:** Principals, administrators, teachers, schools **Temporality:** Ongoing during scale-up	Conduct educational meetings Increase demand

**Table 3:** Baseline values for girls and boys at usual practice (UP) and intervention (INT) schools. Only participants with complete data for the covariates age, BMI and ethnicity are included (see note). Values are mean (SD) or number (%).

	Girls				Boys			
	Total (*n* = 694)	UP (*n* = 340)	INT (*n* = 354)	Difference (95% CI)	Total (*n* = 660)	UP (*n* = 336)	INT (*n* = 324)	Difference (95% CI)
**Age (years)**	9.9 (0.6)	9.9 (0.6)	9.9 (0.6)	0.05 (−0.03, 0.1)	9.9 (0.6)	10.0 (0.6)	9.9 (0.6)	**0.1 (0.03, 0.2)**
**BMI (kg/m^2^)**	18.2 (3.3)	18.3 (3.2)	18.1 (3.5)	0.2 (−0.3, 0.7)	18.8 (3.7) †	18.8 (3.8)	18.9 (3.6)	−0.2 (−0.7, 0.4)
**Maturity offset (years from APHV)**	−1.8 (0.6)	−1.8 (0.6)	−1.9 (0.5)	0.05 (−0.03, 0.1)	−2.9 (0.5) †	−2.9 (0.5)	−3.0 (0.5)	**−0.8 (0.004, 0.1)**
**Ethnicity, # Asian/white/other** **(% Asian/white/other)**	165/394/135 (24/57/19)	110/163/67 (32/48/20)	55/231/68 (16/65/19)	-	173/386/101 (26/58/15)	119/165/52 (35/49/15)	54/221/49 (17/68/15)	-
**PA score (range 1–5)**	3.0 (0.6) *n* = 631	3.0 (0.6) *n* = 328	3.1 (0.6) *n* = 303	−0.1 (−0.2, 0.02)	3.3 (0.7) † *n* = 605	3.2 (0.6) *n* = 326	3.3 (0.7) *n* = 279	−0.1 (−0.2, 0.03)
**MVPA_PAQ_ (min/day)**	101.1 (92.7) *n* = 631	91.4 (81.3) *n* = 328	111.5 (102.8) *n* = 303	**−20.1 (−34.7, −5.5)**	131.8 (112.1) † *n* = 605	120.8 (101.9) *n* = 326	144.6 (121.8) *n* = 279	**−23.8 (−41.9, −5.6)**
**Cardiorespiratory fitness (laps)**	22.6 (11.3) *n* = 571	23.4 (12.0) *n* = 275	21.9 (10.5) *n* = 296	1.4 (−0.4, 3.3)	26.6 (14.6) † *n* = 548	28.9 (15.2) *n* = 286	24.1 (13.5) *n* = 262	**4.8 (2.4, 7.2)**
**Cardiorespiratory fitness (z-score)**	−0.1 (0.8) *n* = 571	−0.1 (0.8) *n* = 275	−0.1 (0.7) *n* = 296	0.1 (−0.04, 0.2)	−0.3 (0.8) † *n* = 548	−0.2 (0.8) *n* = 286	−0.4 (0.7) *n* = 262	**0.2 (0.1, 0.3)**
**Accelerometry**	*n* = 203	*n* = 114	*n* = 89		*n* = 191	*n* = 117	*n* = 74	
**Total PA (counts/min)**	439.4 (126.1)	414.6 (111.7)	471.2 (136.7)	**−56.6 (−91.9, −21.4)**	502.4 (134.6)†	513.5 (133.9)	484.9 (134.7)	28.5 (−10.9, 68.0)
**MVPA_Accel_ (min/day)**	42.3 (17.0)	38.6 (14.8)	47.2 (18.5)	**−8.6 (−13.4, −3.9)**	54.3 (20.1) †	55.6 (20.3)	52.2 (19.8)	3.3 (−2..5, 9.2)
**60 min MVPA/day,** **# (%) meeting/not meeting**	30/173 (15/85)	9/105 (8/92)	21/68 (24/76)	**16%** **(−6, −26)**	66/125 † (35/65)	44/73 (38/62)	22/52 (30/70)	−8% (−22, 6)

UP, Usual practice; INT, intervention; BMI, body mass index; APHV, age at peak height velocity; PA, physical activity; MVPA_PAQ_, Moderate-to-vigorous PA from question 1 of the Physical Activity Questionnaire-Child; MVPA_Accel_, Moderate-to-vigorous PA from accelerometer. Difference calculated as UP-INT; values in bold indicates significant difference between UP and INT within sex; † Significantly different from girls. Note: We excluded 13 students (4 INT girls, 5 INT boys; 1 UP girl, 3 UP boys) from analysis who had medical conditions that interfered with participation in regular PA or cardiovascular health (e.g., cerebral palsy, diabetes, juvenile arthritis, cardiac anomalies, spina bifida). We also excluded 175 participants missing data for one or more covariates: age (6 INT girls, 1 INT boy; 2 UP girls, 3 UP boys), baseline BMI (19 INT girls, 20 INT boys; 42 UP girls, 38 UP boys) or ethnicity (15 INT girls, 7 INT boys; 17 UP girls, 12 UP boys).

**Table 4:** Physical activity delivery (min/week) by teachers at usual practice (UP) and intervention (INT) schools during year 1 and year 2 as determined using weekly activity logs. Values are mean (95% CI).

	UP	INT	Difference (INT−UP)	*p*-Value
Year 1 (*n* = 86)	104.9 (89.2, 120.5)	138.7 (124.5, 152.9)	33.8 (12.7, 54.9)	<0.01
Year 2 (*n* = 79)	110.5 (95.9, 125.1)	124.4 (111.7, 137.1)	13.9 (−5.5, 33.3)	0.2
Difference (Year 2−Year 1)	5.6 (−10.1, 21.3) *p* = 0.5	−14.2 (−29.5, 1.1) *p* = 0.07		

UP, usual practice; INT, intervention.

**Table 5:** Physical activity (PA) and cardiorespiratory fitness (CRF) at the end of year 1 (T2) and 2 (T4) in girls and boys attending usual practice (UP) compared with intervention (INT) schools. Values are means (SD) or adjusted mean difference (95% CI) using an intention to treat analysis. We include effect size (ES; Cohen’s d) as an indicator of the magnitude of the between-group difference.

	Girls				Boys			
	UP	INT	Adjusted Difference (INT-UP) *	ES	UP	INT	Adjusted Difference (INT-UP) *	ES
**PA Score**								
T2	3.1 (0.6) *n* = 214	3.2 (0.6) *n* = 126	0.05 (−0.1, 0.2)	0.08	3.5 (0.6) *n* = 207	3.3 (0.6) *n* = 99	−0.1 (−0.2, −0.03)	−0.16
T4	3.0 (0.6) *n* = 256	3.1 (0.6) *n* = 236	0.02 (−0.1, 0.2)	0.03	3.4 (0.6) *n* = 263	3.3 (0.6) *n* = 193	−0.1 (−0.3, 0.02)	−0.23
**MVPA_PAQ_ (min/day)**								
T2	103.1 (87.7) *n* = 214	124.6 (101.3) *n* = 126	4.8 (−21.5, 31.2)	0.05	150.5 (100.4) *n* = 207	141.8 (101.3) *n* = 99	−10.8 (−35.4, 13.7)	−0.1
T4	77.7 (51.8) *n* = 256	96.9 (72.4) *n* = 236	10.9 (−3.8, 25.6)	0.17	108.1 (67.1) *n* = 263	115.2 (77.9) *n* = 193	2.2 (−16.6, 21.1)	0.04
**Total PA (counts/min)**								
T2	498.2 (181.0) *n* = 84	566.7 (248.3) *n* = 67	1.2 (−73.0, 75.4)	0.01	615.1 (191.9) *n* = 77	588.5 (275.8) *n* = 55	11.1 (−152.4, 174.5)	0.05
T4	471.5 (174.3) *n* = 77	500.7 (185.3) *n* = 56	−38.1 (−108.6, 32.3)	−0.2	567.4 (190.3) *n* = 76	535.6 (169.8) *n* = 44	−6.9 (−109.1, 95.3)	−0.04
**MVPA_Accel_ (min/day)**								
T2	47.3 (19.4) *n* = 84	56.2 (23.1) *n* = 67	−0.05 (−6.1, 6.0)	0.0	70.2 (26.0) *n* = 77	60.2 (26.1) *n* = 55	−4.2 (−18.7, 10.3)	−0.16
T4	46.0 (18.5) *n* = 77	51.9 (22.0) *n* = 56	−1.9 (−8.5, 4.7)	−0.1	64.9 (25.6) *n* = 76	59.8 (23.6) *n* = 44	−1.5 (−17.3, 14.4)	−0.06
**CRF (# laps)**								
T2	27.4 (13.6) *n* = 183	33.8 (18.8) *n* = 98	7.5 (−0.5, 15.5)	0.48	33.8 (16.8) *n* = 172	35.4 (17.6) *n* = 92	4.7 (−1.2, 10.6)	0.28
T4	30.4 (14.2) *n* = 215	28.8 (14.1) *n* = 221	−0.8 (−5.8, 4.1)	−0.06	38.3 (18.0) *n* = 226	34.2 (16.2) *n* = 180	0.5 (−3.9, 5.0)	0.03

MVPA, Moderate to vigorous physical activity; PAQ, Physical Activity Questionnaire for Children; Accel, accelerometer; CRF, cardiorespiratory fitness. * Adjusted difference at follow up (T2 or T4) between UP and INT groups; adjusted for baseline (T1) score, age, BMI, ethnicity and school cluster.

## Data Availability

The datasets used during the current study are not publicly available as stipulated in our participant consent forms but are available from the authors on reasonable request.

## Supplementary Materials

The following are available online at https://www.mdpi.com/article/10.3390/ijerph18105182/s1.

## References

[1] Chaput J.P., Willumsen J., Bull F., Chou R., Ekelund U., Firth J., Jago R., Ortega F.B., Katzmarzyk P.T. (2020). 2020 WHO guidelines on physical activity and sedentary behaviour for children and adolescents aged 5–17 years: Summary of the evidence. Int. J. Behav. Nutr. Phys. Act..

[2] Ortega F.B., Ruiz J.R., Castillo M.J., Sjostrom M. (2008). Physical fitness in childhood and adolescence: a powerful marker of health. Int. J. Obes..

[3] Steele R.M., Brage S., Corder K., Wareham N.J., Ekelund U. (2008). Physical activity, cardiorespiratory fitness, and the metabolic syndrome in youth. J. Appl. Physiol..

[4] Colley R.C., Carson V., Garriguet D., Janssen I., Roberts K.C., Tremblay M.S. (2017). Physical activity of Canadian children and youth, 2007 to 2015. Health Rep..

[5] Cameron C., Craig C.L., Bauman A., Tudor-Locke C. (2016). CANPLAY study: Secular trends in steps/day amongst 5-19 year-old Canadians between 2005 and 2014. Prev. Med..

[6] Knuth A.G., Hallal P.C. (2009). Temporal trends in physical activity: a systematic review. J. Phys. Act. Health.

[7] Tomkinson G.R., Lang J.J., Tremblay M.S. (2019). Temporal trends in the cardiorespiratory fitness of children and adolescents representing 19 high-income and upper middle-income countries between 1981 and 2014. Br. J. Sports Med..

[8] Craig C.L., Shields M., Leblanc A.G., Tremblay M.S. (2012). Trends in aerobic fitness among Canadians, 1981 to 2007–2009. Appl. Physiol. Nutr. Metab..

[9] Jones R.A., Hinkley T., Okely A.D., Salmon J. (2013). Tracking physical activity and sedentary behavior in childhood: a systematic review. Am. J. Prev. Med..

[10] Janz K.F., Dawson J.D., Mahoney L.T. (2000). Tracking physical fitness and physical activity from childhood to adolescence: the muscatine study. Med. Sci. Sports Exerc..

[11] Andersen L.B., Hasselstrom H., Gronfeldt V., Hansen S.E., Karsten F. (2004). The relationship between physical fitness and clustered risk, and tracking of clustered risk from adolescence to young adulthood: eight years follow-up in the Danish Youth and Sport Study. Int. J. Behav. Nutr. Phys. Act..

[12] Telama R., Yang X., Leskinen E., Kankaanpaa A., Hirvensalo M., Tammelin T., Viikari J.S., Raitakari O.T. (2014). Tracking of physical activity from early childhood through youth into adulthood. Med. Sci. Sports Exerc..

[13] Hogstrom G., Nordstrom A., Nordstrom P. (2016). Aerobic fitness in late adolescence and the risk of early death: a prospective cohort study of 1.3 million Swedish men. Int. J. Epidemiol..

[14] Hogstrom G., Nordstrom A., Nordstrom P. (2014). High aerobic fitness in late adolescence is associated with a reduced risk of myocardial infarction later in life: a nationwide cohort study in men. Eur. Heart J..

[15] Hogstrom G., Ohlsson H., Crump C., Sundquist J., Sundquist K. (2019). Aerobic fitness in late adolescence and the risk of cancer and cancer-associated mortality in adulthood: A prospective nationwide study of 1.2 million Swedish men. Cancer Epidemiol..

[16] Naylor P.J., McKay H.A. (2009). Prevention in the first place: schools a setting for action on physical inactivity. Br. J. Sports Med..

[17] International Society for Physical Activity and Health (ISPAH) (2020). Eight Investments that Work for Physical Activity. www.ISPAH.org/Resources.

[18] Fox K.R., Cooper A., McKenna J. (2004). The School and Promotion of Children’s Health-Enhancing Physical Activity: Perspectives from the United Kingdom. J. Teach Phys. Educ..

[19] Reis R.S., Salvo D., Ogilvie D., Lambert E.V., Goenka S., Brownson R.C. (2016). Scaling up physical activity interventions worldwide: stepping up to larger and smarter approaches to get people moving. Lancet.

[20] Naylor P.J., Nettlefold L., Race D., Hoy C., Ashe M.C., Higgins J.W., McKay H. (2015). Implementation of school based physical activity interventions: A systematic review. Prev. Med..

[21] Cassar S., Salmon J., Timperio A., Naylor P.J., van Nassau F., Contardo Ayala A.M., Koorts H. (2019). Adoption, implementation and sustainability of school-based physical activity and sedentary behaviour interventions in real-world settings: a systematic review. Int. J. Behav. Nutr. Phys. Act..

[22] Love R., Adams J., van Sluijs E.M.F. (2019). Are school-based physical activity interventions effective and equitable? A meta-analysis of cluster randomized controlled trials with accelerometer-assessed activity. Obes. Rev..

[23] Durlak J.A., DuPre E.P. (2008). Implementation matters: a review of research on the influence of implementation on program outcomes and the factors affecting implementation. Am. J. Community Psychol..

[24] Lane C., McCrabb S., Nathan N., Naylor P.J., Bauman A., Milat A., Lum M., Sutherland R., Byaruhanga J., Wolfenden L. (2021). How effective are physical activity interventions when they are scaled-up: a systematic review. Int. J. Behav. Nutr. Phys. Act..

[25] Herlitz L., MacIntyre H., Osborn T., Bonell C. (2020). The sustainability of public health interventions in schools: a systematic review. Implement. Sci..

[26] Milat A.J., King L., Newson R., Wolfenden L., Rissel C., Bauman A., Redman S. (2014). Increasing the scale and adoption of population health interventions: experiences and perspectives of policy makers, practitioners, and researchers. Health Res. Policy Syst..

[27] Naylor P.J., Macdonald H., Reed K.E., McKay H. (2006). Action Schools! BC: A Socioecological Approach to Modifying Chronic Disease Risk Factors in Elementary School Children. Prev. Chronic Dis..

[28] Naylor P.J., Macdonald H.M., Zebedee J.A., Reed K.E., McKay H.A. (2006). Lessons learned from Action Schools! BC--an ‘active school’ model to promote physical activity in elementary schools. J. Sci. Med. Sport.

[29] Naylor P.J., Macdonald H.M., Warburton D.E., Reed K.E., McKay H.A. (2008). An active school model to promote physical activity in elementary schools: Action Schools! BC. Br. J. Sports Med..

[30] Reed K.E., Warburton D.E.R., Macdonald H.M., Naylor P.J., McKay H.A. (2008). Action Schools! BC: A school-based physical activity intervention designed to decrease cardiovascular disease risk factors in children. Prev. Med..

[31] Macdonald H.M., Kontulainen S.A., Petit M.A., Beck T.J., Khan K.M., McKay H.A. (2008). Does a novel school-based physical activity model benefit femoral neck bone strength in pre- and early pubertal children?. Osteoporos. Int..

[32] Ahamed Y., Macdonald H., Reed K., Naylor P.J., Liu-Ambrose T., McKay H. (2007). School-based physical activity does not compromise children’s academic performance. Med. Sci. Sports Exerc..

[33] Indig D., Lee K., Grunseit A., Milat A., Bauman A. (2018). Pathways for scaling up public health interventions. BMC Public Health.

[34] McKay H.A., Macdonald H.M., Nettlefold L., Masse L.C., Day M., Naylor P.J. (2015). Action Schools! BC implementation: from efficacy to effectiveness to scale-up. Br. J. Sports Med..

[35] Powell B.J., Fernandez M.E., Williams N.J., Aarons G.A., Beidas R.S., Lewis C.C., McHugh S.M., Weiner B.J. (2019). Enhancing the Impact of Implementation Strategies in Healthcare: A Research Agenda. Front. Public Health.

[36] Wandersman A., Duffy J., Flaspohler P., Noonan R., Lubell K., Stillman L., Blachman M., Dunville R., Saul J. (2008). Bridging the gap between prevention research and practice: The interactive systems framework for dissemination and implementation. Am. J. Community Psychol..

[37] Simmons R., Shiffman J., Simmons R., Fajans P., Ghiron L. (2007). Chapter 1: Scaling up health service innovations: a framework for action. Scaling up Health Service Delivery: From Pilot Innovations to Policies and Programmes.

[38] Day M.E., Strange K.S., McKay H.A., Naylor P.J. (2008). Action schools! BC--Healthy Eating: effects of a whole-school model to modifying eating behaviours of elementary school children. Can. J. Public Health.

[39] Powell B.J., Waltz T.J., Chinman M.J., Damschroder L.J., Smith J.L., Matthieu M.M., Proctor E.K., Kirchner J.E. (2015). A refined compilation of implementation strategies: results from the Expert Recommendations for Implementing Change (ERIC) project. Implement. Sci..

[40] Leeman J., Birken S.A., Powell B.J., Rohweder C., Shea C.M. (2017). Beyond "implementation strategies": classifying the full range of strategies used in implementation science and practice. Implement. Sci..

[41] Proctor E.K., Powell B.J., McMillen J.C. (2013). Implementation strategies: recommendations for specifying and reporting. Implement. Sci..

[42] Curran G.M., Bauer M., Mittman B., Pyne J.M., Stetler C. (2012). Effectiveness-implementation hybrid designs: combining elements of clinical effectiveness and implementation research to enhance public health impact. Med. Care.

[43] Crocker P.R., Bailey D.A., Faulkner R.A., Kowalski K.C., McGrath R. (1977). Measuring general levels of physical activity: preliminary evidence for the Physical Activity Questionnaire for Older Children. Med. Sci. Sports Exerc..

[44] Mackelvie K.J., McKay H.A., Khan K.M., Crocker P.R. (2001). A school-based exercise intervention augments bone mineral accrual in early pubertal girls. J. Pediatr..

[45] Trost S.G., Loprinzi P.D., Moore R., Pfeiffer K.A. (2011). Comparison of accelerometer cut points for predicting activity intensity in youth. Med. Sci. Sports Exerc..

[46] Evenson K.R., Catellier D.J., Gill K., Ondrak K.S., McMurray R.G. (2008). Calibration of two objective measures of physical activity for children. J. Sports Sci..

[47] World Health Organization (2020). WHO Guidelines on Physical Activity and Sedentary Behaviour.

[48] Leger L.A., Mercier D., Gadoury C., Lambert J. (1988). The multistage 20 metre shuttle run test for aerobic fitness. J. Sports Sci..

[49] Olds T., Tomkinson G., Leger L., Cazorla G. (2006). Worldwide variation in the performance of children and adolescents: an analysis of 109 studies of the 20-m shuttle run test in 37 countries. J. Sports Sci..

[50] Moore S.A., McKay H.A., Macdonald H., Nettlefold L., Baxter-Jones A.D., Cameron N., Brasher P.M. (2015). Enhancing a Somatic Maturity Prediction Model. Med. Sci. Sports Exerc..

[51] Ip F. (2008). Ethnicity and Visible Minority Characteristics of BC’s Population. 2006 Census Fast Facts. 2006-12.

[52] Cohen J. (1992). A power primer. Psychol. Bull..

[53] Linden A. (2019). ESIZEREG: Stata Module for Computing the Effect Size Based on a Linear Regression Coefficient.

[54] Sullivan G.M., Feinn R. (2012). Using Effect Size-or Why the P Value Is Not Enough. J. Grad. Med. Educ..

[55] Global Advocacy for Physical Activity (GAPA), The Advocacy Council of the International Society for Physical Activity and Health (ISPAH) NCD Prevention: Investments that work for physical activity. Br. J. Sports Med..

[56] Keshavarz N., Nutbeam D., Rowling L., Khavarpour F. (2010). Schools as social complex adaptive systems: A new way to understand the challenges of introducing the health promoting schools concept. Soc. Sci. Med..

[57] Lee K., van Nassau F., Grunseit A., Conte K., Milat A., Wolfenden L., Bauman A. (2020). Scaling up population health interventions from decision to sustainability - a window of opportunity? A qualitative view from policy-makers. Health Res. Policy Syst..

[58] Neely K.C., Montemurro G.R., Storey K.E. (2020). A Canadian-wide perspective on the essential conditions for taking a comprehensive school health approach. BMC Public Health.

[59] Flaspohler P., Duffy J., Wandersman A., Stillman L., Maras M.A. (2008). Unpacking prevention capacity: an intersection of research-to-practice models and community-centered models. Am. J. Community Psychol..

[60] Leeman J., Calancie L., Hartman M.A., Escoffery C.T., Herrmann A.K., Tague L.E., Moore A.A., Wilson K.M., Schreiner M., Samuel-Hodge C. (2015). What strategies are used to build practitioners’ capacity to implement community-based interventions and are they effective? A systematic review. Implement. Sci..

[61] Johnson S.M. (2019). Where Teachers Thrive: Organizing Schools for Success.

[62] Jones M., Defever E., Letsinger A., Steele J., Mackintosh K.A. (2020). A mixed-studies systematic review and meta-analysis of school-based interventions to promote physical activity and/or reduce sedentary time in children. J. Sport Health Sci..

[63] Hartwig T.B., Sanders T., Vasconcellos D., Noetel M., Parker P.D., Lubans D.R., Andrade S., Avila-Garcia M., Bartholomew J., Belton S. (2021). School-based interventions modestly increase physical activity and cardiorespiratory fitness but are least effective for youth who need them most: an individual participant pooled analysis of 20 controlled trials. Br. J. Sports Med..

[64] Kriemler S., Meyer U., Martin E., van Sluijs E.M.F., Andersen L.B., Martin B.W. (2011). Effect of school-based interventions on physical activity and fitness in children and adolescents: a review of reviews and systematic update. Br. J. Sports Med..

[65] Armstrong N., Welsman J.O. (2020). Traditional and New Perspectives on Youth Cardiorespiratory Fitness. Med. Sci. Sports Exerc..

[66] Blair S.N., Kohl H.W., Barlow C.E., Paffenbarger R.S., Gibbons L.W., Macera C.A. (1995). Changes in physical fitness and all-cause mortality. A prospective study of healthy and unhealthy men. JAMA.

[67] McCrabb S., Lane C., Hall A., Milat A., Bauman A., Sutherland R., Yoong S., Wolfenden L. (2019). Scaling-up evidence-based obesity interventions: A systematic review assessing intervention adaptations and effectiveness and quantifying the scale-up penalty. Obes. Rev..

[68] Welsh B.C., Sullivan C.J., Olds D.L. (2010). When early crime prevention goes to scale: a new look at the evidence. Prev. Sci..

[69] Tommeraas T., Ogden T. (2017). Is There a Scale-up Penalty? Testing Behavioral Change in the Scaling up of Parent Management Training in Norway. Adm. Policy Ment. Health.

[70] Kilbourne A.M., Neumann M.S., Pincus H.A., Bauer M.S., Stall R. (2007). Implementing evidence-based interventions in health care: application of the replicating effective programs framework. Implement. Sci..

[71] Bopp M., Saunders R.P., Lattimore D. (2013). The tug-of-war: fidelity versus adaptation throughout the health promotion program life cycle. J. Prim. Prev..

[72] Yamey G. (2011). Scaling up global health interventions: a proposed framework for success. PLoS Med..

[73] CATCH Global Foundation (2021). How does CATCH work?. https://catchinfo.org/about/.

[74] Kibbe D.L., Hackett J., Hurley M., McFarland A., Schubert K.G., Schultz A., Harris S. (2011). Ten Years of TAKE 10!((R)): Integrating physical activity with academic concepts in elementary school classrooms. Prev. Med..

[75] Wears R.L. (2002). Advanced Statistics: Statistical Methods for Analyzing Cluster and Cluster-randomized Data. Acad. Emerg. Med..

[76] Williams R.L. (2000). A note on robust variance estimation for cluster correlated data. Biometrics.

