# Supplementary material for: Scaling up Action Schools! BC: How Does Voltage Drop at Scale Affect Student Level Outcomes? A Cluster Randomized Controlled Trial
Source: Int J Environ Res Public Health. 2021 May 13;18(10):5182. doi: 10.3390/ijerph18105182 (PMC8153156; doi:10.3390/ijerph18105182)
Supplement: Supplementary file 1 [file ijerph-18-05182-s001.zip › ijerph-1178565-supplementary.pdf]

Supplementary Table S1. Physical activity (PA) and cardiorespiratory fitness (CRF) at the end of year 2 (T4) in girls and boys attending usual practice (UP) compared with intervention (INT) schools. Values are means (SD) or adjusted mean difference (95% CI) using a sensitivity analysis.

	**Girls**			**Boys**		
	**UP**	**INT**	**Adjusted Difference (INT-UP)**	**UP**	**INT**	**Adjusted Difference (INT-UP)**
**PA Score**	3.1 (0.6) n=190	3.1 (0.6) n=302	-0.02 (-0.2, 0.1)	3.4 (0.6) n=214	3.3 (0.6) n=242	-0.2 (-0.3, -0.03)
**$MVPA_{PAQ}$ (min/d)**	77.3 (50.7) n=190	93.0 (69.4) n=302	10.2 (-3.0, 23.4)	108.1 (65.3) n=214	113.7 (77.3) n=242	3.2 (-13.6, 19.9)
**Average PA (counts/min)**	497.2 (187.9) n=53	474.9 (173.3) n=80	-68.4 (-110.4, -26.4)	606.4 (201.0) n=55	512.8 (155.2) n=65	-73.2 (-163.1, 16.8)
**$MVPA_{Accel}$ (min/d)**	47.8 (18.6) n=53	48.9 (21.3) n=80	-5.5 (-10.4, -0.6)	70.1 (26.7) n=55	57.1 (21.7) n=65	-10.6 (-23.7, 2.5)
**CRF (# laps)**	30.4 (14.2) n=215	28.8 (14.1) n=221	2.3 (-2.1, 6.6)	38.3 (18.0) n=226	34.2 (16.2) n=180	3.0 (-1.7, 7.7)

PAQ-C, Physical Activity Questionnaire for Children; PA, physical activity; MVPA, Moderate to vigorous physical activity

*Adjusted difference at follow-up (T4) between UP and INT groups; adjusted for baseline score, age, BMI ethnicity and school cluster.

Supplementary Table S2. The intraclass correlation coefficient (ICC) for student-level outcomes within the randomized controlled trial. We include the ICC at baseline, across year 1 and from baseline to end of year 2.

	**Baseline**	**T1-T2**	**T1-T4**
PA Score	0.05	0.003	0.08
$MVPA_{PAQ}$ (min/day)	0.09	0.05	0.09
Average PA (counts/min)	0.03	0.07	0.10
$MVPA_{Accel}$ (min/day)	0.03	0.05	0.10
CRF (# laps)	0.18	0.20	0.14
CRF (z-score)	0.16	0.20	0.13

Supplementary Table S3. Pearson correlations between Total physical activity (PA) and Classroom Action PA delivered by teachers at intervention (INT) schools and change in outcomes over each academic year in children attending INT schools.

	**Total PA Delivery (min/wk)**	**Classroom Action Delivery (min/wk)**
**PA Score**		
Year 1	0.09	0.05
Year 2	-0.07	-0.06
**$MVPA_{PAQ}$ (min/day)**		
Year 1	**0.17***	0.04
Year 2	0.02	0.02
**Total PA (counts/min)**		
Year 1	-0.02	0.06
Year 2	-0.01	0.03
**$MVPA_{Accel}$ (min/day)**		
Year 1	-0.03	-0.005
Year 2	-0.04	-0.03
**CRF (# laps)**		
Year 1	0.06	-0.14
Year 2	0.06	0.01
**CRF (z-score)**		
Year 1	0.03	-0.12
Year 2	0.05	-0.02

PA, physical activity; MVPA, Moderate to vigorous physical activity
*$p<0.05$

Supplementary Table S4. Change in physical activity (PA) and cardiorespiratory fitness (CRF) outcomes in children receiving low (< 60%) or high (≥60 %) doses of Classroom Action PA. Values are means (SD) or mean difference (95% CI), adjusted for school cluster.

	High Dose (≥60%)	Low Dose (<60%)	Difference (High-Low)	p-value
**PA Score**				
Year 1	0.2 (0.5) n=30	0.1 (0.6) n=145	0.09 (-0.02, 0.2)	0.1
Year 2	-0.03 (0.5) n=68	0.004 (0.6) n=376	-0.03 (-0.2, 0.2)	0.7
**MVPA$_{PAQ}$ (min/day)**				
Year 1	9.4 (145.0) n=30	22.6 (111.7) n=145	-13.2 (-102.2, 75.8)	0.7
Year 2	-2.9 (92.2) n=57	0.2 (83.6) n=307	-2.2 (-32.5, 28.1)	0.9
**Average PA (counts/min)**				
Year 1	167.7 (273.4) n=16	61.5 (169.8) n=88	106.2 (-26.9, 239.2)	0.09
Year 2	4.9 (108.3) n=23	35.1 (132.8) n=70	-30.2 (-115.1, 54.8)	0.3
**MVPA$_{Accel}$ (min/day)**				
Year 1	9.6 (25.2) n=16	7.6 (20.8) n=88	2.0 (-33.5, 37.5)	0.9
Year 2	1.4 (15.0) n=23	6.7 (18.3) n=70	-5.3 (-20.4, 9.8)	0.3
**CRF (# laps)**				
Year 1	16.6 (17.6) n=38	12.4 (14.1) n=130	4.1 (-12.9, 21.1)	0.5
Year 2	1.3 (9.8) n=53	0.9 (10.4) n=309	0.4 (-3.5, 4.3)	0.8
**CRF (z-score)**				
Year 1	0.82 (0.92) n=36	0.64 (0.78) n=129	0.2 (-0.7, 1.0)	0.6
Year 2	-0.11 (0.6) n=53	-0.06 (0.6) n=309	-0.05 (-0.23, 0.13)	0.5
